# Environmental Pollutant Benzo[*a*]Pyrene Impacts the Volatile Metabolome and Transcriptome of the Human Gut Microbiota

**DOI:** 10.3389/fmicb.2017.01562

**Published:** 2017-08-15

**Authors:** Clémence Defois, Jérémy Ratel, Sylvain Denis, Bérénice Batut, Réjane Beugnot, Eric Peyretaillade, Erwan Engel, Pierre Peyret

**Affiliations:** ^1^MEDIS, Institut National de la Recherche Agronomique, Université Clermont Auvergne Clermont-Ferrand, France; ^2^UR370 QuaPA, MASS Team, Institut National de la Recherche Agronomique Saint-Genes-Champanelle, France

**Keywords:** human gut microbiota, polycyclic aromatic hydrocarbons, 16S amplicon sequencing, volatolomics, metatranscriptomics

## Abstract

Benzo[*a*]pyrene (B[*a*]P) is a ubiquitous, persistent, and carcinogenic pollutant that belongs to the large family of polycyclic aromatic hydrocarbons. Population exposure primarily occurs via contaminated food products, which introduces the pollutant to the digestive tract. Although the metabolism of B[*a*]P by host cells is well known, its impacts on the human gut microbiota, which plays a key role in health and disease, remain unexplored. We performed an *in vitro* assay using 16S barcoding, metatranscriptomics and volatile metabolomics to study the impact of B[*a*]P on two distinct human fecal microbiota. B[*a*]P exposure did not induce a significant change in the microbial structure; however, it altered the microbial volatolome in a dose-dependent manner. The transcript levels related to several metabolic pathways, such as vitamin and cofactor metabolism, cell wall compound metabolism, DNA repair and replication systems, and aromatic compound metabolism, were upregulated, whereas the transcript levels related to the glycolysis-gluconeogenesis pathway and bacterial chemotaxis toward simple carbohydrates were downregulated. These primary findings show that food pollutants, such as B[*a*]P, alter human gut microbiota activity. The observed shift in the volatolome demonstrates that B[*a*]P induces a specific deviation in the microbial metabolism.

## Introduction

Polycyclic aromatic hydrocarbons (PAHs) are of great concern as environmental and foodborne pollutants. These persistent organic pollutants are known to remain in environmental compartments (air, water, soil, and food) and bioaccumulate in organisms (Douben, 2003). Benzo[*a*]pyrene (B[*a*]P) is the most well-characterized and toxic member of the PAH compound family. Because of its mutagenic and carcinogenic effects in animal models (Baird et al., 2005; Huderson et al., 2013) and the genotoxic effects observed in humans exposed to B[*a*]P-containing mixtures (White et al., 2016), B[*a*]P has been categorized as a human group 1 carcinogen by the IARC (IARC, 2012). Human exposure to PAHs mainly occurs via oral uptake from naturally contaminated food products (Martorell et al., 2012; Veyrand et al., 2013) and the consumption of charcoal-grilled, roasted or smoked food (Lee et al., 2016). However, a recent Chinese cohort study determined that the contribution of high-PAH containing foods, such as barbecued, smoked or deep-fried meats, was approximately 13% of the overall daily dietary intake because of the low associated consumption rates (Duan et al., 2016). Exposure to PAHs occurs to a lesser extent via the inhalation of polluted air particles from cigarette smoke, engine exhaust and domestic heating. Animal model experiments along with *in vitro* cellular studies and clinical studies have shown that the toxicity of B[*a*]P and B[*a*]P-containing mixtures targets different organs in the body and has the potential to develop adenomas. The location of tumors appears to be related to the route of exposure. Inhalation of B[*a*]P preferentially induces lung cancer (Mumford et al., 1993; Denissenko et al., 1996; Ueng et al., 2010), and oral administration preferentially induces tumors and cancers in the GIT (Sinha et al., 2005; Huderson et al., 2013; Labib et al., 2013), liver (Kroese et al., 2001; Ba et al., 2015) and breast (Guo et al., 2015; White et al., 2016). However, because of the difficulty of assigning a particular toxic effect to a particular route of exposure, most clinical studies are focused on environmental B[*a*]P exposure, including air and oral intake (Su et al., 2014; Tian et al., 2016). Furthermore, the inhalation of polluted air particles also leads to B[*a*]P transport to the GIT via the mucociliary clearance mechanism (Möller et al., 2004; Mutlu et al., 2011). Once in the GIT, B[*a*]P enters the entero-hepatic circulatory system and is metabolized by cytochrome P450-dependent monooxygenases present in intestinal enterocytes and liver hepatocytes, thus leading to the formation of diol-epoxide compounds. These toxic molecules subsequently form B[*a*]P-DNA adducts (DNA binding products) that have been previously characterized as necessary for B[*a*]P-initiated carcinogenesis (van Herwaarden et al., 2009).

Although the human enzyme metabolism of B[*a*]P and its impacts on human health have been well described, studies have not been performed to determine the possible impact of B[*a*]P on microorganisms living in the human GIT. This microbial community, known as the human gut microbiota, is considered a near-organ and plays major roles in human health. Several pathologies are associated with characteristic shifts in the structure of the gut microbiota (also called dysbiosis), such as IBD (Kostic et al., 2014) and extra-digestive disorders, which include metabolic syndromes (e.g., obesity, diabetes, etc.), autoimmune disorders and neurological disturbances (Vijay-Kumar et al., 2010; Chen et al., 2014; Mu et al., 2016; Teng et al., 2016; Vatanen et al., 2016). Gut microbiota dysbiosis can also lead to the emergence of intestinal pathogens naturally present in the GIT such as *Clostridium difficile* (Antharam et al., 2013). Exposure of the murine gut microbiota to xenobiotics leads to perturbations in microbial structures and functions (Zhang L. et al., 2015). Zhang L. et al. (2015) showed that dietary TCDF altered mice gut microbiota by reducing the ratio of *Firmicutes* to *Bacteroidetes*. Furthermore, AhR signaling activation induces dietary TCDF to alter many host metabolic pathways involved in hepatic lipogenesis, gluconeogenesis and glycogenolysis, bacterial fermentation, and amino acid and nucleic acid metabolism. Allais et al. (2015) exposed mice to cigarette smoke for 24 weeks and observed alterations in the bacterial community structure. Furthermore, the level of 16S rRNA expression (and therefore the activity) of *Lachnospiraceae* sp. strongly increased in the colon. Besides metagenomics and metatranscriptomics, volatolomics has proven to be a promising omic approach to diagnose metabolism changes in response to physiological stresses induced by pathology (Hakim et al., 2012) or xenobiotic exposure (Berge et al., 2011). Regarding gastrointestinal or inflammatory disorders, Ahmed et al. (2013, 2016) reported that changes in fecal VOC pattern may result from changes in the microbiota and/or pathologies in the GIT.

Although studies have not focused on the impact of B[*a*]P on the human gut microbiota, B[*a*]P metabolization has been demonstrated by this microbial consortium. van de Wiele et al. (2005) showed, *in vitro*, that the human gut microbiota can biotransform B[*a*]P into estrogenic metabolites, and they also identified 7-hydroxybenzo[*a*]pyrene as a B[*a*]P derivative.

To highlight the underlying mechanisms and associated consequences of the interaction between B[*a*]P and gut microbiota, we performed 16S barcoding, metatranscriptomic and volatolomic analysis to determine the impact of B[*a*]P on two distinct human fecal microbiota.

## Materials and Methods

### Experimental Design

B[*a*]P (≥96% HPLC, Sigma–Aldrich, Saint-Quentin Fallavier, France) was incubated in batches with three replicates along with two different FM suspensions sampled from the continuous fermentor ECSIM (Feria-Gervasio et al., 2014). The FM suspensions contained *in vitro* cultured FM, which were collected from two human volunteer donors: fecal microbiota-1 (FM-1) and fecal microbiota-2 (FM-2) (This study was a non-interventional study with no additions to usual clinical care. According to the French Health Public Law (CSP Art L 1121-1.1), such a protocol does not require approval of an ethics committee). B[*a*]P is a highly hydrophobic organic compound; thus, to avoid solubility issues in next incubation step, B[*a*]P was dissolved in sunflower seed oil (Sigma–Aldrich, Saint-Quentin Fallavier, France) as described in a wide range of animal studies (Verhofstad et al., 2010; Hodek et al., 2013). The B[*a*]P and all contaminated effluents and materials were handled in an advised and safe manner with all necessary precautions.

Both FM-1 and FM-2 were incubated (i) with B[*a*]P (dissolved in sunflower seed oil) at three serial concentrations of 0.005, 0.05, and 0.5 mg/mL, (ii) with sunflower seed oil (vehicle) and (iii) without B[*a*]P or sunflower seed oil (control). The incubation step was performed in amber flasks (to avoid photocatalytic effects) under shaking at 37°C for 24 h. As the incubation step is processed in a batch fermentor, the incubation time cannot go beyond 24 h because of medium nutrient depletion, acidification and cell accumulation. The incubation volume (30 mL) was composed of one-fourth FM (sampled from the continuous fermentor) and three-fourths colon medium (see Supplementary Table S1).

At the beginning (T0) and the end (T24) of the incubation step, samples dedicated to DNA extraction were stored at -20°C and those for RNA extraction were immediately centrifuged at 900 × *g* for 8 min. The pellets were then resuspended in five volumes of RNA*later*^®^ (Fisher Scientific, Illkirch, France) and maintained at -80°C until extraction. The remaining incubation medium was maintained at -80°C for the VOCs analysis.

### Nucleic Acid Extraction

Genomic DNA (gDNA) extractions were performed using a QIAamp DNA Stool Mini Kit (Qiagen, Courtaboeuf, France) with the following modifications: the samples were centrifuged for 7 min at 900 × *g* and 4°C and pellets were resuspended with ASL buffer according to the manufacturer’s instructions. The final elution volume was 120 μL instead of 200 μL. The quantity and quality of the gDNA were assessed using a NanoDrop 2000 spectrophotometer (Thermo Scientific) and by gDNA electrophoresis on a 0.8% agarose gel.

Total RNA extractions were performed using the RNeasy Plus Mini Kit (Qiagen) with the following modifications: the samples were centrifuged for 8 min at 6000 × *g* to promote flow through the RNA*later*^®^ before initiating the cell lysis step. The remaining DNA was removed using a TURBO DNA-*free*^TM^ kit (Fisher Scientific). The quantity and quality of the treated RNA were assessed using a NanoDrop 2000 spectrophotometer and an Agilent 2100 Bioanalyzer (Agilent Technologies, Courtaboeuf, France). Finally, the treated RNA was either stored at -80°C until the RNA-Seq analysis or underwent reverse transcription using a SuperScript^®^ III First-Strand Synthesis System (Fisher Scientific) to generate 16S rRNA-based amplicons for the barcoding analysis.

### 16S rDNA/rRNA Amplicon Sequencing and Analysis

Library construction and paired-end sequencing (2 × 300 bp) were conducted at UMR1289 TANDEM (Toulouse, France) on an Illumina MiSeq platform. The V3–V4 region of the 16S rRNA genes was amplified using the “universal” bacterial primers 343F (ACGGRAGGCAGCAG) (Liu et al., 2007) and 784R (TACCAGGGTATCTAATCCT) (Andersson et al., 2008), and the library construction was assessed using the TruSeq DNA library preparation protocol (Illumina). 16S rDNA/rRNA amplicon sequencing was performed on both gDNA (referred to as 16S rDNA amplicon sequencing) and reverse-transcribed RNA (referred to as 16S rRNA amplicon sequencing). gDNA was used to investigate the presence of bacteria that are dead or alive within the sample, and cDNA from rRNA was used to investigate bacteria that are alive and active within the sample.

Paired-end reads were joined with fastq-join from the ea-utils software package (Aronesty, 2013), and the resulting sequences were analyzed using the software package Quantitative Insights Into Microbial Ecology (QIIME v. 1.8.0) (Caporaso et al., 2010). After de-multiplexing, the sequences were assessed for quality with PRINSEQ (Schmieder and Edwards, 2011), and chimeras were removed using USEARCH 6.1 (Edgar, 2010). Operational taxonomic units (OTUs) were generated with a 97% similarity threshold and taxonomically assigned using the Greengenes database (DeSantis et al., 2006) with UCLUST (Edgar, 2010).

The samples were randomly normalized to the same sequencing depth (16,595 and 10,716 sequences for the FM-1 samples (for the rDNA and rRNA amplicons, respectively), 30,495 and 24,538 sequences for the FM-2 samples (for the rDNA and rRNA amplicons, respectively). The alpha- (Chao1, Shannon index) and beta-diversity (PCoA on a weighted Unifrac distance matrix) analyses were performed using QIIME. Statistical analyses of the microbial relative abundances were conducted using the Mann-Whitney *U*-test with GraphPad Prism 5 software (San Diego, CA, United States), and the statistical significance was set at *p* < 0.05.

### Microbial Volatolome Analysis

The volatile compounds in the samples were analyzed via solid-phase microextraction (SPME) coupled with gas chromatography-mass spectrometry (GC-MS) as previously described (Bouhlel et al., 2017). Briefly, an automated sampler (MPS2, Gerstel) was used to conduct the following successive steps: (i) the sample was preheated in the agitator (500 rpm) for 10 min at 40°C, (ii) the volatile compounds were trapped by SPME (75 μm carboxen-polydimethylsiloxane, 23 gauge needle, Supelco) for 30 min at 40°C, and (iii) thermal desorption was performed at 280°C for 2 min in splitless mode in the GC inlet. A volatile compounds analysis was performed by GC-full scan MS (GC6890, MS5973N, Agilent). The volatile compounds were separated on a RTX-5MS column (60 m × 0.32 mm × 1 μm, Restek) according to previously established settings (Bouhlel et al., 2017). The volatiles were tentatively identified according to a comparison between their mass spectra and the NIST 14 mass spectral library and between published retention indices (RI) values and the RI values of an internal databank. The peak area of the tentatively identified compounds was determined for each of the targeted molecules using a mass fragment selected for its specificity and freedom from co-elution.

The data were processed using the Statistica Software (v.10) (StatSoft, Maisons-Alfort, France) and the R software (v.2.1.4). ANOVAs (*p* < 0.05) with a Dunnett’s *post hoc* test were conducted on the data and principal component analyses (PCA) were performed on the discriminant volatile compounds selected to visualize the structure of the data.

### RNA Sequencing (RNA-Seq) and Analysis

Pooled total RNA (from the three biological replicates) was depleted in the 16S and 23S rRNA using a solution hybridization method (adapted from Ribo-Zero^TM^ rRNA Removal kit). Library construction (following the TruSeq Stranded mRNA Sample Preparation, Illumina) and paired-end sequencing (MiSeq, 2 × 300 bp) were performed at Fasteris (Plan-les-Ouates, Switzerland).

The paired-end sequences were assessed for quality with PRINSEQ (Schmieder and Edwards, 2011) and joined with fastq-join from the ea-utils software package (Aronesty, 2013), and the rRNA sequences were removed from the data set using SortMeRNA (v. 2.0) software (Kopylova et al., 2012). The rRNA depleted-data set was then submitted to a BLASTX analysis with Diamond (Buchfink et al., 2014) against the NCBI non-redundant protein database (nr). Hits with an *e*-value smaller than 1 × 10^-3^ were investigated with the MEGAN software (v5), (Huson et al., 2007) to perform functional assignation using the KEGG classification. The MEGAN software supports, since version4 (Huson et al., 2011) metagenomic and metatranscriptomic data sets and has been used in studies of microbial ecology (Yu and Zhang, 2012). Finally, the reads per kilobase per million mapped reads (RPKM) abundances of the KEGG functional pathways were obtained using the HMP Unified Metabolic Analysis Network2 (HUMAnN2) software (v0.5) (Abubucker et al., 2012).

## Results

### No Significant Changes in the Structure of Human Fecal Microbiota Following B[*a*]P Exposure

Two human FM were exposed to three B[*a*]P concentrations (0.005, 0.05, and 0.5 mg/mL) for 24 h, and their structures were assessed via 16S rDNA/rRNA-based amplicon sequencing. Treatments without B[*a*]P but with sunflower seed oil (vehicle) or without oil (control) were also conducted to differentiate the impact of B[*a*]P on the microbial communities. At T24 for each condition (the three B[*a*]P concentrations, vehicle and control), the results are provided as the mean relative abundances of the three technical replicates. Because all of the conditions started from the same FM suspension, the results at T0 are considered the mean relative abundances of the fifteen technical replicates.

**FIGURE 1:**
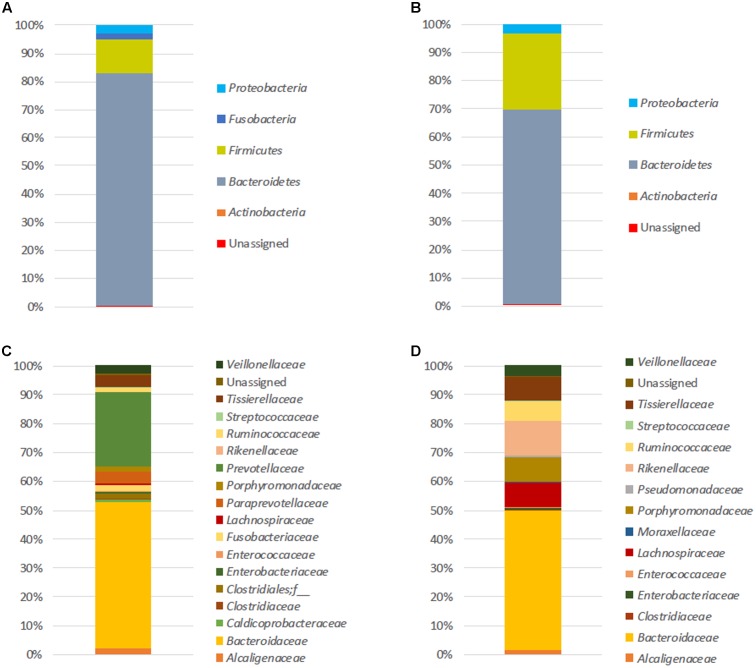
Initial fecal microbiota structures. Relative abundances (%) at the phylum **(A,B)** and family **(C,D)** levels of both FM-1 **(A–C)** and FM-2 **(B–D)** at T0. Analysis based on 16S rDNA amplicon sequencing.

At T0, the number of mean observed OTUs for FM-1 and FM-2 was 250 and 292 for 16S rDNA analysis, respectively, and 151 and 324 for the 16S rRNA analysis, respectively (see Supplementary Table S2). The initial microbial structure (based on the 16S rDNA amplicons) (**Figure 1**) showed that the FM-1 and FM-2 compositions were clearly distinct. Although *Bacteroidetes* and *Firmicutes* were the two most represented phyla found in both structures (82.6 and 12.2% for FM-1 and 69 and 27.1% for FM-2, respectively) (**Figures 1A,B**), the dominant family compositions differed (**Figures 1C,D**). Indeed, the *Bacteroidaceae* and *Prevotellaceae* families strongly dominated the FM-1 structure and showed 50.7 and 26% relative abundances, respectively, whereas *Bacteroidaceae* (48.1%), *Rikenellaceae* (12.2%), *Porphyromonadaceae* (8.8%), *Lachnospiraceae* (8.4%), *Tissierellaceae* (7.9%), and *Ruminococcaceae* (7%) were the most represented families in the FM-2 structure. The most represented OTUs were assigned to *Bacteroides uniformis* (34.1% of sequences) and *Bacteroides* sp. (33.8% of sequences) for FM-1 and FM-2, respectively. The 16S rRNA-based amplicon analysis (see Supplementary Figure S1) presented differences in the composition of the active microbiota, even at the phylum level (see Supplementary Figures S1A,B). Although the phylum *Bacteroidetes* is represented in both structures with the same relative abundance (60–65%), the microbial activity in FM-1 and FM-2 was dominated by the phyla *Fusobacteria* (28%) and *Firmicutes* (26.9%), respectively. The differences increased at the family level (see Supplementary Figures S1C,D), with three dominant active families for FM-1 [*Prevotellaceae* (29.8%), *Fusobacteriaceae* (28%), and *Bacteroidaceae* (25.5%)] and four for FM-2 [*Bacteroidaceae* (31.5%), *Ruminococcaceae* (21.8%), *Rikenellaceae* (17.8%), and *Porphyromonadaceae* (15.4%)]. The most represented OTUs were assigned to *Prevotella copri* (28% of sequences) and *Bacteroides* sp. (20.4% of sequences) for FM-1 and FM-2, respectively.

At T24 and depending on the experimental conditions, the number of observed OTUs for FM-1 ranged from 341 to 373 for the 16S rDNA analysis and from 147 to 190 for the 16S rRNA analysis. For FM-2, the number of observed OTUs ranged from 242 to 309 for the 16S rDNA analysis and from 264 to 310 for the 16S rRNA analysis (see Supplementary Table S2). In the batch cultures, dead cells were not removed from the medium; therefore, after 24 h of fermentation, the DNA extracted from the medium could not be used to determine the structure of the microbial community, and only the 16S rRNA sequences were used to analyze and compare the FM structures.

**FIGURE 2:**
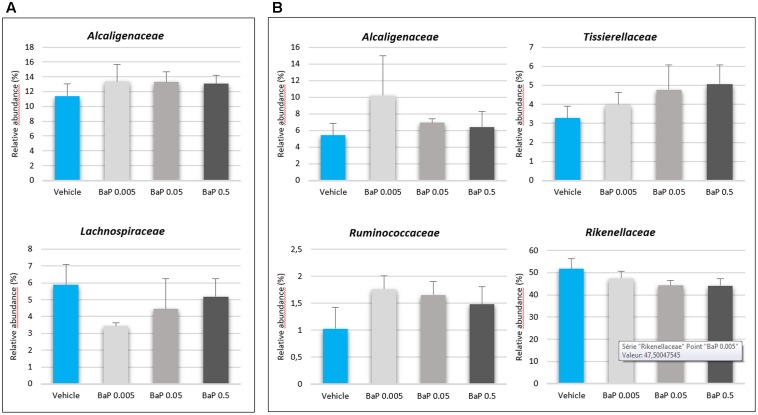
Variations in the microbiota composition following B[*a*]P exposure. Relative abundances (%) of the active bacterial families modulated by 24 h of B[*a*]P exposure at 0.005, 0.05, or 0.5 mg/mL in FM-1 **(A)** and FM-2 **(B)**. Values are shown as the mean ± SEM. A Mann–Whitney *U*-test showed no significant differences.

For both FM, significant differences were not observed in the alpha-diversity among the vehicle, control and B[*a*]P exposed samples. The diversity (Shannon’s index) and richness (chao1, observed_otus) values were equivalent for each condition tested (see Supplementary Table S2). A PCoA shows that sunflower seed oil exposure induced a change in the fecal bacterial pattern compared to the control samples (without B[*a*]P nor oil) (see Supplementary Figure S2). This result confirms the need to include a vehicle control in such an experimental design as observed in other studies (Maurice et al., 2013; Zhang L. et al., 2015). Thus, to remove the sunflower oil effects, the B[*a*]P conditions were further directly compared with vehicle samples. After 24 h of incubation, significant changes were not observed in the structure of the active FM after B[*a*]P exposure (see Supplementary Figure S3). However, relative abundance trends occurred at the family level (**Figure 2**), as the increase in the abundance of the *Alcaligenaceae* family for both microbiota primarily at the lowest dose of B[*a*]P (0.005 mg/mL). The mean relative abundance increased from 11.4 to 13.4% and from 5.4% to 12.2% for FM-1 and FM-2, respectively. The *Ruminococcaceae* and *Tissierellaceae* families also increased from 1.0 to 1.8% for FM-1 (at 0.005 mg/mL) and from 3.3 to 5.1% (at 0.5 mg/mL) for FM-2. Finally, the *Lachnospiraceae* (from 5.9 to 3.4% at 0.005 mg/mL) and the *Rikenellaceae* (5.2 to 4.4% at 0.5 mg/mL) families decreased for FM-1 and FM-2, respectively.

### B[*a*]P Exposure Alters the Microbial Volatolome

Although B[*a*]P did not significantly perturb the FM structures, the microbial activity could be altered. Microbial VOCs are the result of microbial biological activity. The volatolome may thus reflect deviations in microbial metabolism in response to a stimulus. In this work, a non-targeted analysis of the microbial volatolome was conducted to detect potential volatile biomarkers as a response to B[*a*]P exposure.

**FIGURE 3:**
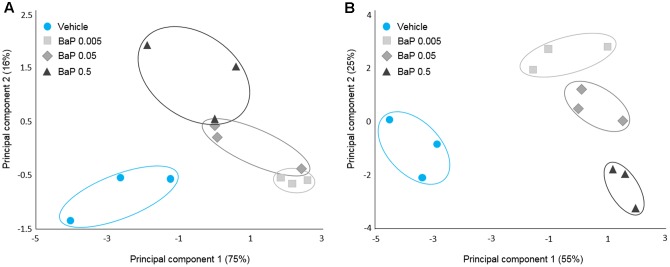
Microbial volatolome patterns following B[*a*]P exposure. Principal component analysis (PCA) of the six potential markers (volatile organic compounds) of B[*a*]P exposure for FM-1 **(A)** and the 16 potential markers of B[*a*]P exposure for FM-2 selected by ANOVA (*p* < 0.05) **(B)**. Triplicates for each condition are shown.

To characterize the effect of B[*a*]P on the microbial volatolome, more than 200 VOCs were determined by SPME-GC-MS after the incubation step (T24). PCAs were performed on the discriminant VOCs selected by ANOVA with Dunnett’s *post hoc* test. To account for the sunflower seed oil effect, the B[*a*]P results were compared with the vehicle sample results. For each microbiota (FM-1 and FM-2), vehicle samples were clearly differentiated from the three B[*a*]P conditions, and each B[*a*]P condition was distinct from the others (**Figure 3**). Furthermore, B[*a*]P exposure appears to change the microbial volatolome in a dose-dependent manner because a linear trend was observed between the three dose groups.

To determine the combined FM responses under B[*a*]P exposure, an ANOVA with Dunnett’s *post hoc* test was performed on the combined data set of the two microbiota. The **Table 1** lists the seven VOCs which are detected in the microbiota volatolome as significantly altered by the exposure to B[*a*]P, among which six out of them have been tentatively identified. These shared markers highlight similar functional responses for the two microbiota in terms of volatolome, even if the two microbiota structures were clearly different as shown in **Figure 1**.

**Table 1:** Volatile metabolites detected in the volatolome of the two fecal microbiota as significantly altered by B[*a*]P exposure compared to control.

Volatile metabolite^1^	m/z	LRI^2^	Peak abundance (×10^3^)			
			
			Vehicle	BaP 0.005	BaP 0.05	BaP 0.5
Benzaldehyde	106	971	510.8	456.1	527.0	604.4^∗^
3-octanone	99	986	64.0	33.9^∗^	39.8^∗^	46.9^∗^
2-pentylfuran	138	992	43.6	32.1	24.8^∗^	18.0^∗^
Butyl butanoate	57	994	292.4	142.2^∗^	149.7^∗^	171.0^∗^
Unknown	99	1032	7.3	3.1^∗^	3.3^∗^	3.4^∗^
2-methyl-phenol	108	1049	9.6	8.1	10.1	13.6^∗^
2-hexylfuran	81	1087	23.0	17.9	15.7	14.8^∗^


*^1^Tentative identification based on mass spectrum and LRI from literature or internal databank. ^2^Linear retention indices on a DB5 capillary column. ^∗^Significance (*p* < 0.05) according to Dunnett’s *post hoc* test (control: vehicle).*

### B[*a*]P Exposure Alters the Microbial Metatranscriptome

The volatolome analysis showed that the microbial activity changed after 24 h of B[*a*]P exposure. Thus, we investigated the microbial gene response. The FM metatranscriptome analysis was performed on the samples exposed to the highest concentration of B[*a*]P (0.5 mg/mL) at T24 to identify how B[*a*]P exposure affected the transcript levels. A functional assignation of metabolic pathways was performed using the KEGG classification, and the results are given as RPKM abundances. To avoid sunflower seed oil effect, the B[*a*]P samples were directly compared with the vehicle samples. More than 200 metabolic KEGG pathways derived from the KEGG Orthology (KO) database were mapped to the samples (206 and 200 for FM-1 and 240 and 235 for FM-2 for the B[*a*]P and vehicle samples, respectively).

**FIGURE 4:**
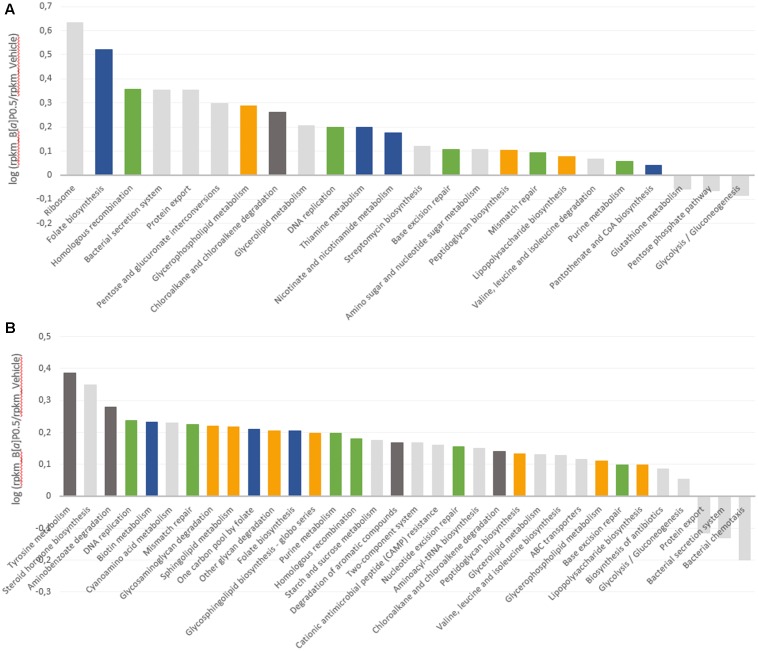
Modulation of the microbial transcript levels following B[*a*]P exposure. KEGG metabolic pathways that are upregulated and downregulated at T24 by B[*a*]P exposure at 0.5 mg/mL are shown in the FM-1 **(A)** and FM-2 **(B)** metatranscriptomes. Variations are expressed as the log of B[*a*]P and vehicle RPKM abundance ratios. Blue: vitamin and cofactor metabolism; orange: cell wall compound metabolism; green: DNA repair and replication systems; and dark gray: aromatic compound metabolism.

Upregulated and downregulated pathways are represented in **Figure 4** as the log of the B[*a*]P and vehicle RPKM abundance ratios. Four metabolic groups (including several KEGG metabolic pathways) that were over-expressed in both metatranscriptomes emerged from the analysis. These pathways included vitamin and cofactor metabolism, cell wall compound metabolism, DNA repair and replication systems, and aromatic compound metabolism. Few pathways involved in carbohydrate metabolism were downregulated by B[*a*]P exposure for both FM. Glycolysis/gluconeogenesis and the pentose phosphate pathway were repressed in the FM-1 metatranscriptome, whereas bacterial chemotaxis toward simple carbohydrate (glucose and galactose) was repressed in the FM-2 metatranscriptome.

## Discussion

B[*a*]P is a ubiquitous environmental pollutant that bioaccumulates in organisms and is formed *de novo* by certain food processing methods, such as smoking and high cooking temperatures. Although digestive and extra digestive pathologies have been linked to compositional or functional alterations of the gut microbiota, B[*a*]P-induced perturbations of the human gut microbiota have not been previously studied.

The B[*a*]P dosages chosen for this work were informed by two previous studies carried out on the interaction between the B[*a*]P and the gut microbiota allowing us to make comparisons and discussions (van de Wiele et al., 2005; Ribière et al., 2016). These doses are higher than the typical daily consumption of B[*a*]P; however, B[*a*]P exposure is chronic throughout a person’s life. The experiment included two clearly distinct FM structures which might reflects the large individual gut microbiota variations found in the human population. Thus similar responses of the two FM toward the B[*a*]P exposure could not be attributed to a restricted microbial composition.

Surprisingly, B[*a*]P did not induce a significant change in the microbial structure, although slight modifications were observed at the family level, such as increases in the *Sutterellaceae* (formerly *Alcaligenaceae*), *Tissierellaceae* and *Ruminococcaceae* families and reductions in the *Rikenellaceae* and *Lachnospiraceae* families. Our laboratory previously investigated the impact of 28 days of oral B[*a*]P exposure on the fecal and intestinal mucosa-associated bacteria in mice (Ribière et al., 2016). Similar to the results of the present work, we showed that B[*a*]P did not have a significant impact on the bacterial richness (Shannon index) and diversity (chao1 index, observed_otus) during the course of exposure. However, we showed that B[*a*]P induced significant shifts in the composition and relative abundance of the stool and mucosa associated bacterial communities. These results differed from those presented here because significant variations in the bacterial structure were not observed, which might have been caused by various aspects of the experimental design. Murine models are widely used in gut microbiota studies; however, discrepancies between human and mice gut bacterial composition have been reported. Although *Bacteroidetes* and *Firmicutes* are the two major phyla dominating the human and mouse gut microbiota (Eckburg et al., 2005; Ley et al., 2005), 85% of the bacterial genera found in mouse gut microbiota are not present in humans (Ley et al., 2005). Finally, several environmental factors may shape the mice gut microbiota, specifically cage effects (including coprophagy), dietary factors (standardized chow diet composed mainly of plant materials), and genetic background (including inbreeding to preserve genetic homogeneity).

Similarly to our present work, Maurice et al. (2013) showed that a short period of exposure (4 h) with six host-targeting drugs did not alter the microbial active community structure of the human gut microbiota. A prior work from Sawulski et al. (2014) demonstrated that in the soil environment, alterations in the bacterial community structure may be correlated to the pattern of degradation of a given PAH. B[*a*]P was degraded much more slowly than less complex PAHs and to a lower extent, which was reflected in slow changes of the bacterial community structure (no significant variations were observed after 2 days of exposure). Changes of the gut microbiota structure after xenobiotic exposure has previously been reported, and most studies in which xenobiotic exposure (antibiotics excluded) altered the mice microbial structure lasted at least several days and often weeks (Breton et al., 2013; Chassaing et al., 2015; Zhang Y. et al., 2015; Ribière et al., 2016). Thus, to test such a chronic exposure, a 28-days experiment in continuous fermentor will be carried out in the laboratory.

Although the two FM structures appeared to remain stable after a short exposure time, the bacterial activity may have adapted to the presence of the toxic compound. The volatolome profiling has been rapidly emerging in disease diagnosis (Arasaradnam et al., 2014), and changes in VOC patterns have been reported in many physiological and pathological states, including gut dysbiosis in gastrointestinal diseases. Seven VOCs were found to have significantly altered concentrations in both FMs tested under exposure to B[*a*]P (**Table 1**). There are no reported links between these compounds and known microbial metabolic processes. As end-products, these compounds may arise from diverse microbial metabolic pathways. Control samples with the pollutant but without gut microbiota were assessed in the SPME-GC-MS analysis. VOCs that are identified as shifting in the manuscript were either not found in control samples or were present in control samples but showed no abundance variation between vehicle and B[*a*]P samples. We did not look at the fungi community. However, using the SortmeRNA software (Kopylova et al., 2012) on the RNA-Seq data, we did not see any ribosomal eukaryote sequences. Thereby, excluding an abundance below the detection limit, VOCs seem to arise from prokaryote activity.

The depletion of volatolome esters like butyl butanoate has recently been reported in response to gastrointestinal metabolic disorders (De Preter et al., 2015). Accordingly, Ahmed et al. (2015) suggested that differences in levels of fecal esters may reveal changes in the fermentation process of the gut microbiota. Regarding changes in benzaldehyde, 2-methyl-phenol, 3-octanone, 2-pentylfuran, 2-hexylfuran, De Preter et al. (2013) reported that the depletion or augmentation of metabolites belonging to benzenoids, ketones and furan derivatives would probably be a consequence of the disruption of the normal bacterial ecology in pathologies like IBD. It could be hypothesized that these same compounds would also trigger a gut metabolic response to chemical agents such as B[*a*]P.

In the present study, B[*a*]P exposure alters the gut microbiota volatolome in a dose-dependent manner. Furthermore, although the bacterial composition of the two studied microbiota were clearly different, their metabolic responses were similar with seven markers significantly altered by B[*a*]P exposure in both microbiota. This shift in the volatolome shows that B[*a*]P induces a deviation in the microbial metabolism. At present, no systematic studies have been conducted on the identification of gut microbiota volatolome after xenobiotic exposure. Nonetheless, Berge et al. (2011) noted a shift in the volatolome of liver cells of chickens exposed to various dietary chemical contaminants, including a mixture of three PAHs among which was B[*a*]P. Therefore, the present work may enlarge again the field of potential applications of volatolomics, which already includes cancer diagnosis (Hakim et al., 2012; Broza et al., 2015), food authentication (Engel et al., 2007; Sivadier et al., 2008), and ecotoxicology (Ratel and Engel, 2009; Berge et al., 2011).

Finally, to further characterize the influence of B[*a*]P on the active gut microbiota, we analyzed gene expression using RNA sequencing. Transcripts related to four KEGG metabolic pathways were found to be upregulated in both fecal metatranscriptomes. These pathways include vitamin and cofactor metabolism, cell wall compound metabolism, DNA repair and replication systems, and aromatic compound metabolism.

Some of these results may be linked to previous data from the literature as for the lipophilic and mutagenic properties of the B[*a*]P. As lipophilic compounds, PAHs have been shown to penetrate into the cytoplasmic membrane, which results in an increase in membrane fluidity that leads to the loss of membrane functionality and bacterial cell damage (Sikkema et al., 1994). One of the major adaptive mechanisms of bacteria cells to counteract this effect is to increase membrane rigidity (enhance membrane lipid saturation) to prevent compound accumulation (Murínová and Dercová, 2014). This bacterial physiological adaptation may explain the increase observed in the level of transcripts implicated in the metabolism of cell wall compounds. The mutagenic effect of B[*a*]P is also known to occur via DNA adducts (Baird et al., 2005). DeMarini et al. (2011) characterized the mutagenic potencies and mutation spectra of B[*a*]P in *Salmonella* strains TA98, TA100, and TA104. The results from this study showed that (i) 24% of the mutations induced by B[*a*]P in *Salmonella* led to complex frameshifts, (ii) B[*a*]P produced primarily GC to TA transversions and (iii) the majority (96%) of stable adducts induced were at a guanine site. Similarly, the mutagenic potencies of the B[*a*]P might explain the observed induction of the DNA repair and replication systems. Furthermore, within the vitamin and cofactor metabolism pathway, the transcript levels related to folate biosynthesis, which is a major cofactor in DNA synthesis (Harvey and Dev, 1975; Sangurdekar et al., 2011), increased in both metatranscriptomes.

In the FM-2 metatranscriptome, transcripts related to the steroid hormone biosynthesis pathway were also upregulated, which is consistent with prior work from van de Wiele et al. (2005). They implemented a batch fermentation system and showed the ability of the human gut microbiota to metabolize several PAHs, including B[*a*]P, to metabolites with estrogenic activity. Indeed, several hydroxylated-PAH metabolites are structurally similar to the steroidal hormones that bind the human estrogen receptor (ER) (Fertuck et al., 2001; Hirose et al., 2001; Sievers et al., 2013). These authors confirmed the PAH biotransformation by the identification of the metabolites 1-hydroxypyrene and 7-hydroxybenzo[*a*]pyrene in colon digests of pyrene and B[*a*]P, respectively. Finally, few pathways were downregulated, such as the glycolysis-gluconeogenesis and the bacterial chemotaxis toward simple carbohydrates. This reduction in energy metabolism may be part of the cell adaptation process. Stressful environmental conditions, such as B[*a*]P exposure, may lead the cells to rapidly perform coping mechanisms. Microorganisms may thus engage energy for adaptation mechanisms to ensure necessary physiological functions prior to devoting resources to energy consumption for growth and multiplication (Murínová and Dercová, 2014). The ability of gut microbial strains to bind B[*a*]P via physical adsorption to peptidoglycans has also been reported (Hongfei et al., 2013). Thus, another hypothesis is that steric hindrance caused by B[*a*]P adsorption limits the bacterial ability to sense carbohydrates in its environment.

## Conclusion

For the first time, a model of acute B[*a*]P exposure in the human gut microbiota was initiated. Although significant impacts were not observed on the gut microbiota structure, the microbial activity was altered both at the metatranscriptome and at the volatolome levels that could contribute to disturb human gut homeostasis. Humans are exposed to B[*a*]P as well as to mixtures of PAHs and other environmental and foodborne chemicals. Thus, our results suggested that further works should consider pollutants that could contribute to the development of microbial functional dysbiosis, which will potentially impair the human host homeostasis.

## Accession Codes

All sequence data produced via 16S rRNA/rDNA amplicon and RNA sequencing are available in the NCBI Sequence Read Archive, BioProject PRJNA339203, under accession no. SRP082261.

## Author Contributions

CD, PP, EP, and EE conceived the study and design the experiments. CD and JR performed experimental procedures. CD, JR, EE, and PP analyzed data. SD provided support for batch fermentations and RB for metatranscriptomic experiments. BB helped with treatments of sequencing data analysis. CD, PP, EP, and EE interpreted data and wrote the manuscript. All authors reviewed and approved the manuscript. All authors agree to be accountable for all aspects of the work.

## Conflict of Interest Statement

The authors declare that the research was conducted in the absence of any commercial or financial relationships that could be construed as a potential conflict of interest.

## Abbreviations

AhR: aryl hydrocarbon receptor
B[*a*]P: benzo[*a*]pyrene
ECSIM: environmental control system for intestinal microbiota
FM: fecal microbiota
GIT: gastrointestinal tract
IARC: International Agency for Research on Cancer
IBD: inflammatory bowel disease
PAH: polycyclic aromatic hydrocarbon
PCoA: principal coordinate analysis
TCDF: 2,3,7,8-tetrachloro dibenzofuran
VOC: volatile organic compound
