# Supplementary material for: Environmental Pollutant Benzo[a]Pyrene Impacts the Volatile Metabolome and Transcriptome of the Human Gut Microbiota
Source: Front Microbiol. 2017 Aug 15;8:1562. doi: 10.3389/fmicb.2017.01562 (PMC5559432; doi:10.3389/fmicb.2017.01562)
Supplement: Supplementary file 1 [file Data_Sheet_1.PDF]

Supplementary Material

ENVIRONMENTAL POLLUTANT BENZO[*A*]PYRENE IMPACTS THE VOLATILE METABOLOME AND TRANSCRIPTOME OF THE HUMAN GUT MICROBIOTA

Clémence Defois[1], Jérémy Ratel[2], Sylvain Denis[1], Bérénice Batut[1], Réjane Beugnot[1], Eric Peyretaillade[1], Erwan Engel[2] and Pierre Peyret[1*]

* Corresponding author: Pierre Peyret
Tel: +33 (0)473178308; E-mail: pierre.peyret@udamail.fr

PREPARATION FOR :	1 L	UNIT
Tween 80	1	g
Demineralized water	990	mL
Reach 50°C with microwave heating		
Incorporate with a Turrax homogenizer:		
Potato starch	4	g
Corn starch	1	g
Cellulose	2	g
Pectin	2	g
Xylan	1	g
Arabinogalactan	2	g
Arabic gum	0.67	g
Guar	0.33	g
Inulin	1	g
Primatone	5	g
Bacto tryptone	5	g
Bacto Peptone	3	g
Yeast extract	4	g
Mucin type II	4	g
Soy lecithin	375	mg
Egg yolk	125	mg
Bile salts	200	mg
NaH2PO4 2H2O	19.4	g
K2HPO4 3H2O	5.8	g
NaCl	4.5	g
KCl	4.5	g
MgSO4 7H2O	200	mg
FeSO4 7H2O solution (2.5 $g.L^{-1}$)	2	mL
Hemin solution (5 $g.L^{-1}$)	1	mL
L-cystein-HCl	500	mg
Resazurin solution (1 $g.L^{-1}$)	1	mL
Ajust pH at 6.2		
CaCl2 2H2O	100	mg

VITAMIN SOLUTION	$mg.L^{-1}$
Menadione	1
D-biotin	2
Panthothenate	10
Nicotinamide	5
Vitamine B_{12}	0.5
Thiamin	4
Para-aminobenzoic acid	5

Supplementary Table S1. Media composition and preparation for batch fermentation. After homogenizing all of the media components, the media were flushed with nitrogen *via* shaking (500 rpm) and heating (75 °C) on a Labotech EM3300T. Under nitrogen flow, 22 mL of media was aliquoted into 50-mL vials and autoclaved at 115 °C for 15 min. Before the incubation period (T0), 1 µL of a vitamin solution was added to each 50-mL vial along with the FM suspension sampled from the continuous fermentor ECSIM (7.5 mL) and either the B[*a*]P-sunflower seed oil solution (0.5 mL) or the sunflower seed oil alone (0.5 mL). The final fermentation volume was 30 mL.

FM-1

		T0			T24		
		Shannon	chao1	observed_otus	Shannon	chao1	observed_otus
**a**	BaP 0.005				4.25 ± 0.04	645.3 ± 70.3	349.7 ± 12.5
	BaP 0.05				4.28 ± 0.09	744.2 ± 147.0	373.3 ± 17.9
	BaP 0.5	3.57 ± 0.34	411.3 ± 104.9	250.1 ± 64.9	4.35 ± 0.16	670.0 ± 72.0	357.3 ± 32.4
	CT				4.06 ± 0.05	596.4 ± 18.2	341.3 ± 11.8
	Vehicle				4.27 ± 0.18	667.2 ± 105.8	354.3 ± 34.9
**b**	BaP 0.005				3.37 ± 0.04	315.9 ± 76.4	163.7 ± 10.7
	BaP 0.05				3.47 ± 0.10	311.8 ± 38.3	182.5 ± 0.7
	BaP 0.5	3.07 ± 0.18	247.1 ± 64.5	151.3 ± 28.4	3.51 ± 0.19	289.8 ± 11.7	157.0 ± 1.4
	CT				2.83 ± 0.09	269.4 ± 10.2	147.3 ± 5.8
	Vehicle				3.54 ± 0.15	325.5 ± 40.9	190.0 ± 23.0

FM-2

		T0			T24		
		Shannon	chao1	observed_otus	Shannon	chao1	observed_otus
**a**	BaP 0.005				3.60 ± 0.03	514.8 ± 107.5	285.3 ± 24.8
	BaP 0.05				3.65 ± 0.09	511.3 ± 64.2	300.0 ± 26.6
	BaP 0.5	3.57 ± 0.04	566.4 ± 77.7	291.9 ± 21.5	3.65 ± 0.10	499.4 ± 47.4	299.3 ± 21.9
	CT				3.36 ± 0.16	447.5 ± 23.6	241.7 ± 13.7
	Vehicle				3.72 ± 0.06	516.6 ± 54.9	309.3 ± 9.3
**b**	BaP 0.005				3.20 ± 0.08	514.4 ± 16.2	293.3 ± 14.2
	BaP 0.05				3.30 ± 0.02	594.7 ± 90.9	307.7 ± 3.9
	BaP 0.5	3.71 ± 0.12	574.6 ± 99.2	323.6 ± 50.7	3.34 ± 0.13	591.4 ± 44.4	310.3 ± 40.5
	CT				3.31 ± 0.34	485.4 ± 217.4	279.0 ± 111.4
	Vehicle				3.01 ± 0.19	486.4 ± 46.4	263.7 ± 28.0

Supplementary Table S2. Diversity and richness values for the fecal microbiota samples at T0 and T24 for each condition studied. Diversity (Shannon) and richness (chao1, observed_otus) values were derived from 16S rDNA (a) or 16S rRNA (b) amplicon sequencing. At T24 for each condition, the results are listed as the mean ± SD of the relative abundances of the three biological replicates. Because all of the conditions started from the same FM suspension, the results at T0 are listed as the mean ± SD of the relative abundances of the fifteen biological replicates (5 conditions and three replicates). The operational taxonomic units (OTUs) were defined with 97% sequence identity. Calculations were performed based on rarefied OTU tables with 16,595 and 10,716 sequences for the FM-1 samples (for the rDNA and rRNA amplicons respectively) and 30,495 and 24,538 sequences for the FM-2 samples (for the rDNA and rRNA amplicons respectively).

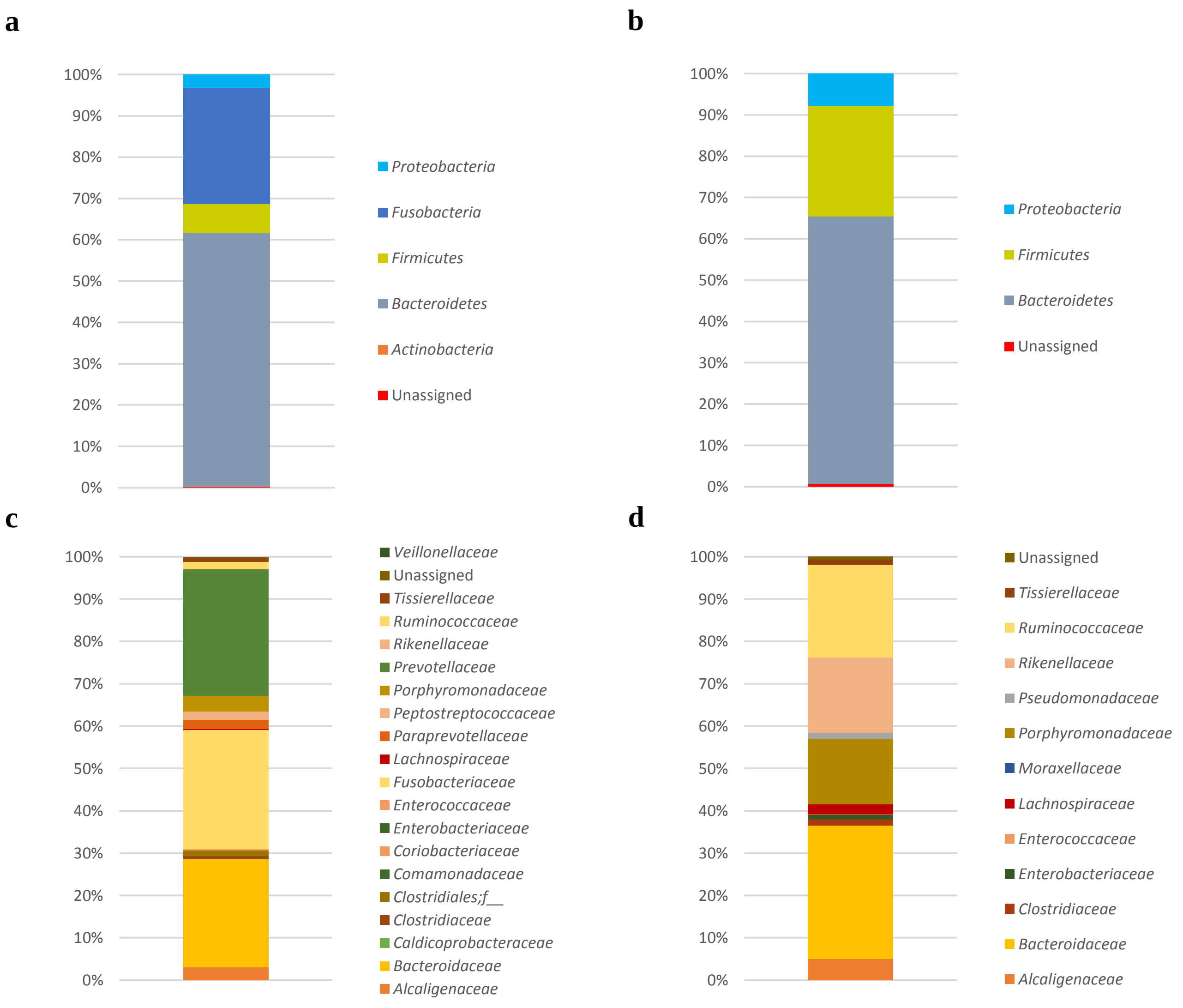

Supplementary Figure S1. Initial structure of the active microbial community. Relative abundances (%) at the phylum (**a-b**) and family (**c-d**) levels of FM-1 (**a-c**) and FM-2 (**b-d**) at T0. Analysis based on 16S rRNA amplicon sequencing.

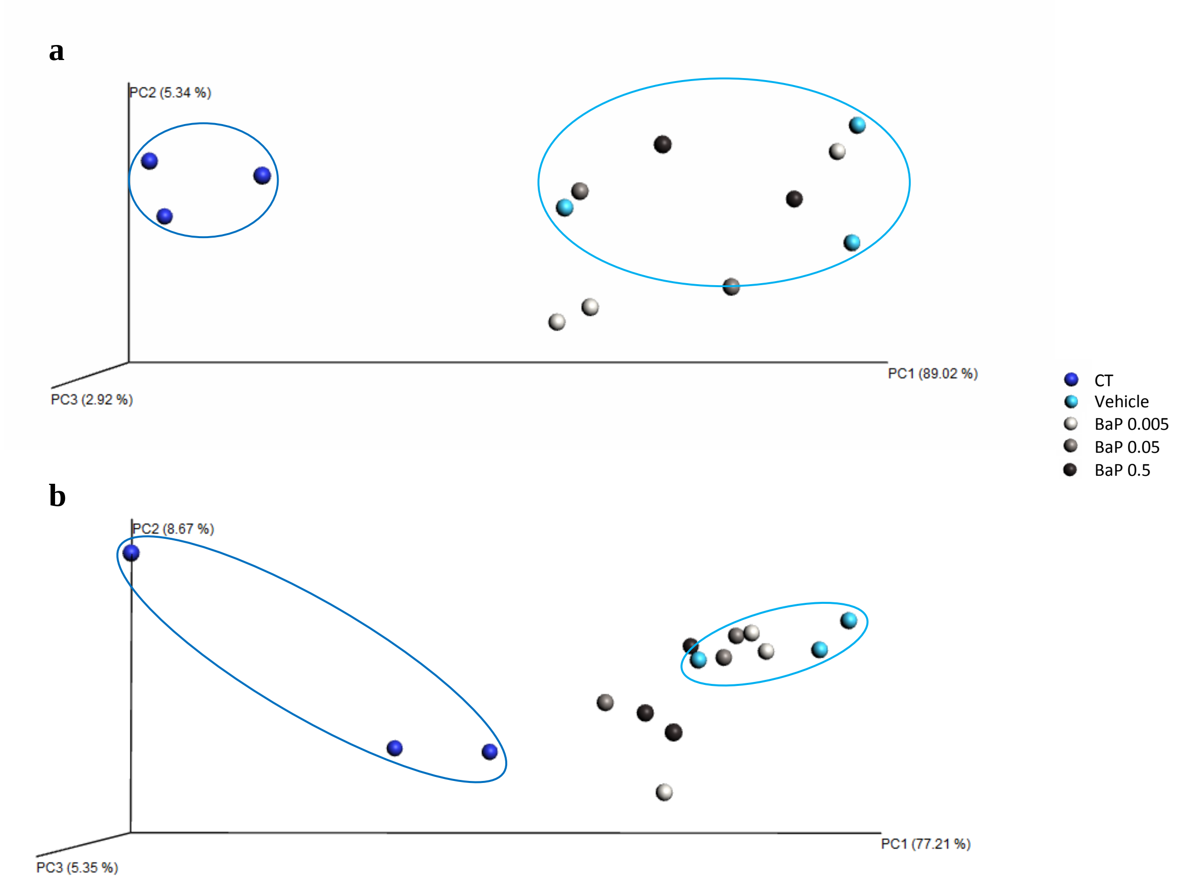

Supplementary Figure S2. Fecal bacterial patterns following B[*a*]P exposure. FM-1 (**a**) and FM-2 (**b**) structures after 24 hr of B[*a*]P exposure; the values are differentiated by a principal coordinate analysis (PCoA) on a weighted UniFrac distance matrix. Analysis based on 16S rRNA amplicon sequencing. The *p*-values from ADONIS were 0.013 (R^2=0.769) and 0.007 (R^2=0.666) for FM-1 and FM-2 respectively.

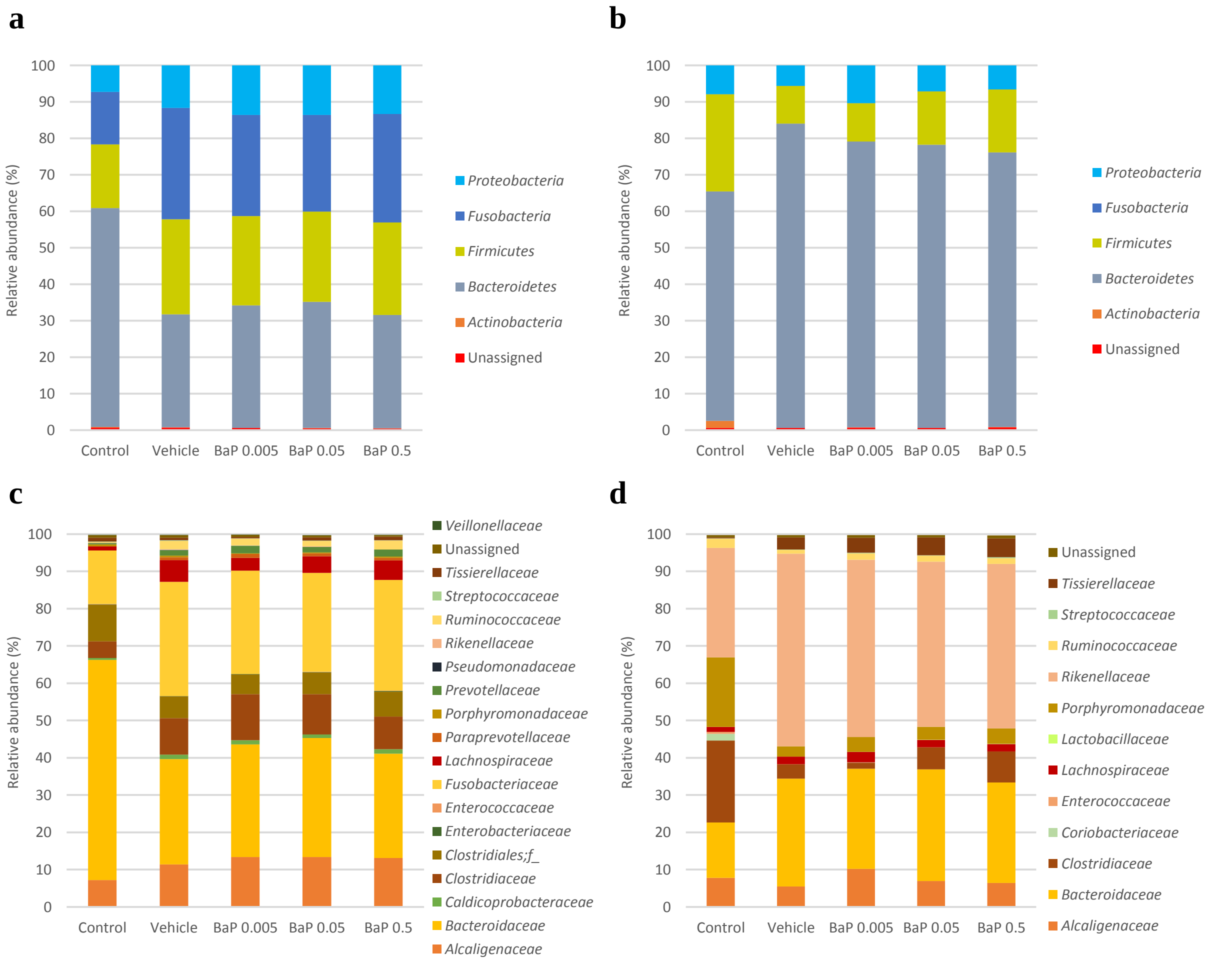

Supplementary Figure S3. Structure of the active microbial community following B[*a*]P exposure. Relative abundances (%) at the phylum (**a-b**) and family (**c-d**) levels of both FM-1 (**a-c**) and FM-2 (**b-d**) at T24. Analysis based on 16S rRNA amplicon sequencing. A Mann-Whitney *U*-test showed no significant differences.
